# Variable- and person-centered approaches to affect-biased attention in infancy reveal unique relations with infant negative affect and maternal anxiety

**DOI:** 10.1038/s41598-021-81119-5

**Published:** 2021-01-18

**Authors:** Alicia Vallorani, Xiaoxue Fu, Santiago Morales, Vanessa LoBue, Kristin A. Buss, Koraly Pérez-Edgar

**Affiliations:** 1 Department of Psychology, The Pennsylvania State University, University Park, PA 16802 USA; 2 University of South Carolina, Columbia, SC USA; 3 University of Maryland, College Park, MD USA; 4 Rutgers University, Newark, NJ USA

**Keywords:** Psychology, Human behaviour

## Abstract

Affect-biased attention is an automatic process that prioritizes emotionally or motivationally salient stimuli. Several models of affect-biased attention and its development suggest that it comprises an individual’s ability to both engage with and disengage from emotional stimuli. Researchers typically rely on singular tasks to measure affect-biased attention, which may lead to inconsistent results across studies. Here we examined affect-biased attention across three tasks in a unique sample of 193 infants, using both variable-centered (factor analysis; FA) and person-centered (latent profile analysis; LPA) approaches. Using exploratory FA, we found evidence for two factors of affect-biased attention: an *Engagement* factor and a *Disengagement* factor, where greater maternal anxiety was related to less engagement with faces. Using LPA, we found two groups of infants with different patterns of affect-biased attention: a *Vigilant* group and an *Avoidant* group. A significant interaction noted that infants higher in negative affect who also had more anxious mothers were most likely to be in the *Vigilant* group. Overall, these results suggest that both FA and LPA are viable approaches for studying distinct questions related to the development of affect-biased attention, and set the stage for future longitudinal work examining the role of infant negative affect and maternal anxiety in the emergence of affect-biased attention.

## Introduction

Infants preferentially attend to faces from the first days of life^1,2^, with emotional faces providing the earliest and most consistent conduit of socioemotional information. However, with development, idiosyncratic systemic biases in how children attend to their socioemotional environment may emerge and become rigid and entrenched^3,4^, leading to what researchers have called affect-biased attention. Affect-biased attention is an automatic process that prioritizes stimuli that are emotionally or motivationally salient to an individual^5^ and relies on orienting to (engagement) and from (disengagement) environmental stimuli^6^. Although both the ability to engage with and disengage from emotional stimuli underpin affect-biased attention, most research assessing affect-biased attention, including our own^7–9^, has relied on individuals completing a single task that may not capture both components well. From that single task, we typically then accept a single mean value or difference score as a metric of an individual’s affect-biased attention, despite agreement that it is a construct that involves multiple attentional processes.

Comparisons of different attention tasks *across studies* have failed to provide consistent results. Emerging research suggests that patterns of affect-biased attention across tasks *within individuals* may better capture relations with anxiety and anxiety risk^10,11^. Thus, it is important for researchers to examine relations between multiple attentional mechanisms captured by different tasks within a single sample^12^. However, because little work has collected data across multiple tasks within a large sample of participants, how to best model relations across tasks is still an open question. It is also not clear if different analytic approaches may reveal different relations with individual difference and contextual factors known to relate to affect-biased attention. The current study leverages a large sample of infants from ages 4 to 24 months who completed a set of affect-biased attention tasks to (1) examine how person- and variable-centered approaches detect systematic patterns of attention and (2) test if these novel patterns are associated with known markers of socioemotional risk, namely temperamental negative affect and maternal anxiety.

The use of singular tasks runs contrary to our theories regarding how affect-biased attention both functions^5^ and develops^3,4^ and may lead to discrepant findings across studies. Indeed, when considering affect-biased attention to threat specifically, research using a single task has found that both a bias toward threat^13–16^ and a bias away from threat^17–20^ are related to anxiety and fearful temperament. Additional work has shown that patterns of attention across tasks, rather than a specific bias towards or away from threat, are related to anxiety^11^ and fearful temperament^10^. In particular, it appears that the stability of attention patterns, rather than the directionality (towards vs away) of attention, is most clearly linked to socioemotional profiles. Thus, examining performance across tasks may better clarify how patterns of affect-biased attention are related to individual difference factors. The current study draws on three tasks designed to capture distinct, but overlapping, components of attention bias.

One of the most common measures of affect-biased attention in adults and children is the dot-probe task^9,13^. The task typically presents two faces (emotion-neutral, neutral-emotion, or neutral–neutral) on opposite sides of the screen. Once the faces disappear, a probe appears on either side of the screen. Depending on task timing, the dot-probe task can capture automatic orienting to a target face (i.e., reaction time to congruent versus incongruent probes) when faces are presented very quickly or even subliminally^21,22^, or engagement with faces (dwell to faces) and disengagement from faces (latency to probes) when the faces are presented for longer periods of time^9^.

Work in young children using very short presentation times suggests that children four years and younger show a general automatic bias for emotion faces rather than a specific bias to either angry or happy faces^22^. Our own previous work in infants 4- to 24-months using longer presentation times indicates that older infants dwell longer to emotion faces (angry and happy) than neutral faces, with slightly longer dwell times to angry faces. Additionally, younger infants low in negative affect who spend more time dwelling to angry faces are faster to then disengage from angry faces to fixate on probes^9^. Thus, the dot-probe task may be sensitive to both age-related changes in both automatic processing of and engagement with emotion faces, as well as temperamental influences on disengagement from emotional stimuli.

The overlap task^8^ is a second affect-biased attention task designed specifically for infant research. In the task, a single face (emotion or neutral) is presented in the center of the screen alone for 1000 ms at which point a probe appears in the left or right visual field. The face and probe are presented simultaneously for 3000 ms^8,23^. Dwell time to faces in the presence of the probe captures engagement with faces in spite of the presence of novel cue that could draw attention. Latency to fixate to or sustained dwell to the probe captures disengagement from faces. Previous work using this task has shown that between the ages of 5- and 7-months, infants begin to exhibit a “stickiness” in their attention to fearful faces such that they both spend more time looking at, and exhibit more difficulty disengaging from, fearful faces^23,24^. However, this bias appears to taper off before 24-months of age^25^. Our own work using this task suggests a bias towards emotion faces, compared to neutral faces in infancy (4–24 months). Moreover, a bias toward angry faces, but not happy faces, was related to greater maternal anxiety^8^. Thus, the overlap task may be sensitive to a normative curvilinear relation between age and engagement with threatening faces during infancy as well as the association with maternal anxiety.

Vigilance tasks, which measure rapid attention to emotional or personally meaningful stimuli, are a third type of affect-biased attention task. Previous work indicates that children and adults are faster to locate a single threatening face in a display of non-threatening faces than a single non-threatening face in a display of threatening faces^26^. Further, 9- to 12-month infants orient faster to angry faces compared to happy faces^27^. Our recently designed vigilance task (see Fu et al., 2020 for task visualization) assesses orienting to faces in the absence of distractors. The task presents a single face (angry, happy or neutral) in random locations at the edges of the visual field. The faces disappear as soon as a fixation occurs. Latency to orient to the face captures initial engagement with faces. Using this task, we found that older infants high in both negative affect and attentional control were faster to orient to neutral faces, rather than emotional faces^7^. Neutral faces are ambiguous relative to angry and happy faces and may draw the attention of infants sensitive to novelty, uncertainty, and ambiguity. Indeed, research has suggested that fearful temperament is associated with discomfort in uncertain social situations^28^. The vigilance task might thus capture how a combination of overcontrolled and fearful temperaments shape rigid attention patterns and risk for anxiety^29^.

In assessing multiple tasks, the first question often centers on how to best integrate the information streams. A variable-centered approach, such as factor analysis (FA), can enable researchers to examine if there are distinct components of affect-biased attention that can be differentiated across multiple tasks. For example, the attention bias literature argues that observed patterns of attention are due to either initial reactive attention to a salient stimulus (engagement) or an inability to shift attention away from a salient stimulus once attended to (disengagement) or a combination of the two^5^. Should FA reveal such individual components, it is then possible to examine how developmental, individual difference and contextual factors separately relate to the factors that make-up affect-biased attention.

Conversely, person-centered approaches, such as latent profile analysis (LPA), can capture profiles of affect-biased attention marked by how engagement and disengagement both cluster within individuals. For example, the clinical and temperament literatures argue that individuals with^13^ or at increased risk^14,16^ for anxiety should show stable patterns of attention towards threat, or in some cases, such as Post-Traumatic Stress Disorder^30^, extreme avoidance of threat. If this expectation is correct, groups of infants that show a vigilant pattern of affect-biased attention (cluster of engagement and disengagement components marking a bias toward emotional cues) could be distinguished from an avoidant pattern of affect-biased attention (cluster of engagement and disengagement components marking a bias away from emotion cues). Researchers could then examine what developmental, individual difference or contextual factors relate to membership in one affect-biased attention group versus another. Both variable- and person-centered approaches to analyzing data are viable with the availability of multiple measures and may answer distinctly different questions despite retaining the same underlying metrics across analytic methods^12^.

Our models of affect-biased attention suggest that individual difference factors present in early life, such as negative affect, may interact with normative developmental changes in attentional capacities and contextual risk factors, such as maternal anxiety, to increase affect-biased attention^3,4^. As described above, there are likely normative changes with development (as captured by the proxy variable of chronological age) that impact the course of affect-biased attention development^9,24,25^. Orienting abilities that are developing throughout the first years of life^6^ may underlie changes in how infants engage with and disengage from affective stimuli.

Negative affect and maternal anxiety may also moderate how the developmental course of affect-biased attention unfolds. Temperamental negative affect, often characterized in the first months of life as distress, anger, and later sadness, can be measured in infants as young as 4-months^31^. Signs of fearfulness emerge as part of the constellation of negative affect in the second half of the first year of life, building on more sophisticated cognitive and socioemotional processing of the environment^32^. Both the broader construct of temperamental negative affect, and the more specific presentation of temperamental fear, have been linked to affect-biased attention^9^. Specific dimensions of temperament may develop in tandem with attention systems^33^, biasing individual interpretations of affective salience. Overcontrolled patterns of attention that emerge in the context of specific temperament profiles may exacerbate anxiety risk^29^.

Maternal anxiety may further moderate relations between age, negative affect, and affect-biased attention. Infants of anxious mothers exhibit increases in negative affect from 9- to 18-months^34^. Additionally, maternal anxiety is associated with infant affect-biased attention both concurrently^8^ and over time^35^. Of course, without a genetically-informed sample design we cannot disentangle the mechanisms by which maternal anxiety impacts infant functioning^34^. However, the available data suggest that maternal anxiety influences how children interpret and interact with their world, either through shared genetic load, children modeling anxiogenic behaviors displayed by their mothers, or a likely combination of both developmental mechanisms^36^.

In the current study we examined affect-biased attention in infants 4- to 24-months of age across three tasks (dot-probe, overlap, vigilance). We had two aims. First, we explored both a variable-centered approach (exploratory factor analysis; FA) and a person-centered approach (latent profile analysis; LPA) to model affect-biased attention. We asked (1) Are there specific components of affect-biased attention across our three tasks (FA)? and (2) Are there groups of infants that exhibit particular types of affect-biased attention (LPA)? Second, we considered how age, negative affect, and maternal anxiety relate to components of affect-biased attention (variable-centered approach) versus how the same three factors relate to being an infant with a particular type of affect-biased attention (person-centered approach). These approaches answer different, but complementary, questions.

Across methods, we anticipated that greater age would be related to metrics suggesting greater attentional control (more engagement with faces and better disengagement from distractors), reflecting developmental change in attentional mechanisms^6^. Because we did not know what factors or groups would emerge from our exploratory FA and LPA respectively, we could not make explicit predictions regarding how negative affect and maternal anxiety might moderate relations between age and affect-biased attention. However, based on the previous literature, we explored individual contributions of negative affect and maternal anxiety, as well as potential interaction effects, on affect-biased attention. Particularly, we anticipated that maternal anxiety and negative affect were likely to be associated with the same factor or profile with prior work showing the mutually reinforcing effects of negative affect and maternal anxiety^34^ and the roles of both temperament^7,9–11^ and maternal anxiety^8,35^ in affect-biased attention.

## Results

**Figure 1:**
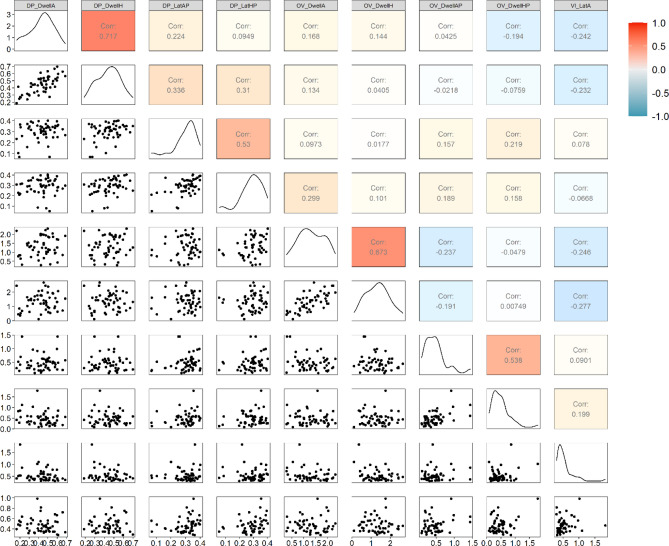
Correlations between eye-tracking metrics of interest. *DP *dot probe task, *OV *overlap task, *VI *vigilance task, *A *angry face, *H *happy face, *AP *probe associated with angry face, *HP *probe associated with happy face.

Table S1 displays descriptive statistics and Fig. 1 displays correlations between our eye-tracking metrics of interest. Descriptive statistics indicated variability in infant attention to the emotion faces. Additionally, our eye-tracking metrics of interest were correlated but not perfectly overlapping, suggesting that we were able to capture different aspects of attention.

### Aim 1: examine variable-centered and person-centered approaches

#### Factor analysis

We conducted an exploratory FA to examine for core components of affect-biased attention across our three tasks. We selected BIC, CFI, SRMR, and RMSEA as our fit measures^37^. We compared 1-, 2- and 3-factor models. In all cases, modification indices suggested modeling the residual covariance between angry and happy faces within the dot-probe task. Thus, we included this residual covariance in all models. A Chi-Squared Difference Test comparing the three models indicated both the 2-factor and 3-factor models were better fitting than the 1-factor model (*p*’s < 0.001). The 2-factor and 3-factor models were not significantly different from each other (*p* = 0.149; Table 1). Thus, we more closely examined the fit statistics for the 2- and 3-factor models (Table S2). The BIC indicated the 2-factor model was the best fit, whereas the SRMR indicated the 3-factor model was a better fit. The CFI and RMSEA did not meaningfully differentiate between the models. We ultimately selected the 2-factor model for further analysis for parsimony, the fact that the individual factors were more clearly disambiguated and its match to theoretical understandings of affect-biased attention.

**Table 1:** 1-, 2- and 3-Factor models assessing affect-biased attention across tasks.

	*b*	*β*	*SE*	*z-value*	*p*
**Two-factor model**					
Factor 1					
DP: dwell time to angry faces	1.00	0.38			
DP: dwell time to happy faces	0.97	0.35	0.22	4.42	0.000
OV: dwell time to angry faces	9.00	0.74	2.38	3.77	0.000
OV: dwell time to happy faces	11.71	0.94	3.10	3.78	0.000
VI: latency to angry faces	− 1.21	− 0.23	0.57	− 2.11	0.034
VI: latency to happy faces	− 1.29	− 0.30	0.55	− 2.37	0.018
Factor 2					
DP: latency to probe in angry trials	1.00	0.70			
DP: latency to probe in happy trials	1.13	0.74	0.32	3.50	0.000
OV: dwell time to probe in angry trials	2.98	0.46	1.16	2.57	0.010
OV: dwell time to probe in happy trials	1.70	0.36	0.70	2.44	0.015
**Three factor model**					
Factor 1					
DP: dwell time to angry faces	1.00	0.38			
DP: dwell time to happy faces	0.99	0.36	0.22	4.45	0.000
OV: dwell time to angry faces	9.11	0.76	2.46	3.70	0.000
OV: dwell time to happy faces	11.43	0.93	2.99	3.83	0.000
Factor 2					
VI: latency to angry faces	1.00	0.35			
VI: latency to happy faces	1.05	0.45	0.51	2.07	0.039
Factor 3					
DP: latency to probe in angry trials	1.00	0.69			
DP: latency to probe in happy trials	1.13	0.74	0.31	3.62	0.000
OV: dwell time to probe in angry trials	3.02	0.46	1.15	2.62	0.009
OV: dwell time to probe in happy trials	1.75	0.37	0.71	2.45	0.014

N = 193. *SE* standard error.

In the 2-factor model, Factor 1 included metrics measuring engagement with faces (dwell time in the dot-probe and overlap tasks and latency in the vigilance task). Thus, we labeled Factor 1 *Engagement*. Importantly, the components of the *Engagement* factor were inversely related meaning longer dwells to faces were coupled with shorter latencies to faces. Higher levels of the *Engagement* factor represent more attention to faces. Factor 2 included metrics measuring disengagement (latency to probe in the dot-probe task and dwell to the probe in the overlap task). We labeled Factor 2 *Disengagement*. Importantly, the components of the *Disengagement* factor were related in the same direction meaning longer latencies to probes (longer to disengage from face to probe) were coupled with longer dwells to probes. Therefore, higher levels of the *Disengagement* factor represent more difficulty disengaging from one stimulus to attend to another rather than a specific difficulty disengaging from faces. The *Engagement* and *Disengagement* factors were unrelated (*b* = 0.001, *p* = 0.172).

#### Latent profile analysis

To examine if there were groups of infants that shared patterns of affect-biased attention, we conducted an LPA. We selected the BIC and BLRT as our primary fit measures as they are superior indicators of LPA model fit compared to other common fit indices, including AIC and entropy^38^; see Table S3 for all fit statistics). In mclust, a larger BIC value indicates better fit as it identifies the model with the greatest integrated likelihood^39^. We compared 1-, 2-, 3- and 4-profile models. The BLRT indicated the 2-profile was significantly better than a 1-profile model (*p* = 0.001) and that the 3-profile model was significantly better than the 2-profile model (*p* = 0.038). A 4-profile model was not significantly better than the 3-profile model (*p* = 0.488). Thus, we more closely examined the means and BICs for the 2- and 3-profile models (Table 2). The BIC indicated that the 2-profile model was a better fit than the 3-profile model. Additionally, all but one (DP: Latency to Probe in Angry Trial) out of our 10 indicators were significantly different between groups in the 2-profile model, whereas multiple indicators were not significantly different between groups in the 3-profile model. We ultimately selected the 2-profile model for further analysis as the groups appeared more distinct and interpretable than the groups identified by the 3-profile model.

**Table 2:** 2- and 3-Profile solutions for LPA assessing affect-biased attention group membership.

	2-Profile solution			3-Profile solution			*f*
	*M1* (*SD1*) N = 147	*M2* (*SD2*) N = 46	*t*	*M1* (*SD1*) N = 36	*M2* (*SD2*) N = 44	*M3* (*SD3*) N = 113	
DP: dwell time to angry faces	0.43 (0.13)	0.34 (0.17)	3.23*	0.54 (0.09)	0.36 (0.16)	0.38 (0.13)	24.64*
DP: dwell time to happy faces	0.43 (0.14)	0.31 (0.16)	4.56*	0.54 (0.10)	0.32 (0.16)	0.38 (0.13)	18.05*
DP: latency to probe in angry trials	0.30 (0.08)	0.33 (0.09)	− 1.70	0.34 (0.03)	0.34 (0.08)	0.28 (0.09)	19.50*
DP: latency to probe in happy trials	0.29 (0.09)	0.33 (0.11)	− 2.60*	0.33 (0.04)	0.34 (0.10)	0.27 (0.10)	18.64*
OV: dwell time to angry faces	1.44 (0.62)	0.81 (0.64)	5.87*	1.22 (0.61)	0.83 (0.64)	1.49 (0.62)	12.28*
OV: dwell time to happy faces	1.51 (0.62)	0.79 (0.68)	6.44*	1.25 (0.69)	0.79 (0.69)	1.59 (0.58)	17.80*
OV: dwell time to probe in angry trials	0.37 (0.27)	0.67 (0.54)	− 3.59*	0.40 (0.29)	0.69 (0.54)	0.36 (0.27)	3.43
OV: dwell time to probe in happy trials	0.35 (0.20)	0.55 (0.40)	− 3.17*	0.31 (0.16)	0.53 (0.41)	0.37 (0.22)	0.00
VI: latency to angry faces	0.50 (0.18)	0.75 (0.47)	− 3.41*	0.55 (0.14)	0.75 (0.48)	0.49 (0.19)	5.05*
VI: latency to happy faces	0.47 (0.13)	0.79 (0.35)	− 6.00*	0.46 (0.12)	0.80 (0.35)	0.48 (0.14)	2.67

N = 193; *p < .05.

*DP *dot-probe task, *OV *overlap task, *VI *vigilance task, *M *mean, *SD *standard deviation.

In the 2-profile model, Group 1 exhibited more time engaging with faces (dwell time) across both the dot-probe and overlap tasks. Additionally, infants were faster to orient to new information across the dot-probe and vigilance tasks (latency to probe and latency to face respectively). Infants also spent less time engaging with the probe (dwell time) during the overlap task. Group 2 exhibited less time engaging with faces and were slower to orient to new information. Thus, in our 2-profile model we deemed Group 1 our *Vigilant* affect-biased attention group and Group 2 our *Avoidant* affect-biased attention group. Importantly, our LPA captured different combinations of attentional mechanisms than our FA.

The *Engagement* factor and the *Disengagement* factors were unrelated within our FA. However, within the *Vigilant* group infants showed both more attention to faces and greater capacity to disengage. Thus, our person-centered approach provided us unique patterns of attention that could not be ascertained by our variable-centered approach.

In addition to determining group membership (dichotomous), LPA reveals the (continuous) *probability* of group membership^40^. For the purposes of Aim 2, we extracted probability of membership in the *Vigilant* group (*M*_Prob_ = 0.74, *SD*_Prob_ = 0.39, *Range*_Prob_ = 0.00 to 1.00; see Table 3 for correlations between Probability of Vigilant Group Membership and LPA variables).

**Table 3:** Correlations between LPA variables and probability of vigilant group membership.

	Probability of vigilant group
DP: dwell time to angry faces	0.26**
DP: dwell time to happy faces	0.39***
DP: latency to probe in angry trials	− 0.07
DP: latency to probe in happy trials	− 0.12
OV: dwell time to angry faces	0.43***
OV: dwell time to happy faces	0.46***
OV: dwell time to probe in angry trials	− 0.38***
OV: dwell time to probe in happy trials	− 0.36***
VI: latency to angry faces	− 0.39***
VI: latency to happy faces	− 0.57***

**p < .01, ***p < .001.

*DP *dot-probe task, *OV *overlap task, *VI *vigilance task.

### Aim 2: examine individual difference and contextual factors in variable-centered and person-centered approaches

#### Structural equation model

To assess how age, negative affect, and maternal anxiety relate to core components of affect-biased attention tasks we regressed the three measures and all potential interactions on our *Engagement* and *Disengagement* factors (Table 4). We found a main effect of maternal anxiety on *Engagement* such that more maternal anxiety was related to less engagement with faces. Additionally, we found a main effect of age on *Disengagement* such that greater age was related to more difficulty disengaging. Negative affect was not associated with either factor and no interactions were noted. We compared this model to a model controlling for infant sex and gestational age at birth via a Chi-Squared Difference Test and found no difference between the models (*p* = 0.095).

**Table 4:** Structural equation model assessing relations between age, negative affect, maternal anxiety and the engagement and disengagement factors.

	*b*	*β*	*SE*	*z*	*p*
**Latent variables**					
Engagement					
DP: dwell time to angry faces	1.00	0.38			
DP: dwell time to happy faces	0.95	0.34	0.21	4.59	0.000
OV: dwell time to angry faces	8.82	0.72	2.26	3.90	0.000
OV: dwell time to happy faces	12.26	0.97	3.34	3.67	0.000
VI: latency to angry faces	− 1.12	− 0.21	0.54	− 2.09	0.037
VI: latency to happy faces	− 1.34	− 0.31	0.53	− 2.55	0.011
Disengagement					
DP: latency to probe in angry trials	1.00	0.73			
DP: latency to probe in happy trials	1.01	0.70	0.33	3.11	0.002
OV: dwell time to probe in angry trials	2.41	0.39	1.27	1.90	0.058
OV: dwell time to probe in happy trials	1.59	0.35	0.73	2.17	0.030
**Regressions**					
Engagement					
Age	0.00	0.14	0.01	0.14	0.886
Negative affect	0.01	0.06	0.01	0.71	0.480
Maternal anxiety	− 0.02	− 0.33	0.01	− 2.72	0.007
Age × negative affect	− 0.01	− 0.18	0.01	− 1.78	0.075
Age × maternal anxiety	0.01	0.12	0.01	1.19	0.235
Negative affect × maternal anxiety	0.01	0.15	0.01	1.19	0.234
Age × negative affect × maternal anxiety	0.00	0.04	0.01	0.43	0.667
Disengagement					
Age	0.02	0.28	0.01	2.09	0.037
Negative affect	− 0.01	− 0.05	0.01	− 0.46	0.648
Maternal anxiety	0.00	0.05	0.01	0.42	0.672
Age × negative affect	0.00	0.03	0.01	0.20	0.841
Age × maternal anxiety	− 0.00	− 0.04	0.01	− 0.27	0.788
Negative affect × maternal anxiety	0.01	0.05	0.01	0.33	0.739
Age × negative affect × maternal anxiety	− 0.00	− 0.02	0.01	− 0.15	0.882

N = 193; *SE* = Standard Error; RMSEA = 0.03; SRMR = 0.07.

#### Multiple regression model

Table 5 displays descriptive statistics and correlations between the variables of interest.

**Table 5:** Descriptive statistics and correlations LPA model.

	2	3	4	5	6	*M*	*SD*	N
1. Sex	0.04	− 0.10	0.13	0.08	− 0.04			193
2. Prematurity		− 0.11	0.03	− 0.01	0.04	− 2.86	10.00	192
3. Age			0.04	− 0.10	0.07	12.34	5.67	193
4. Negative affect				0.14	0.04	− 0.05	0.70	192
5. Maternal anxiety					− 0.06	4.67	5.59	168
6. Probability of vigilant group membership						0.74	0.39	193

No relations were significant at the *p* < 0.05 level.

**Figure 2:**
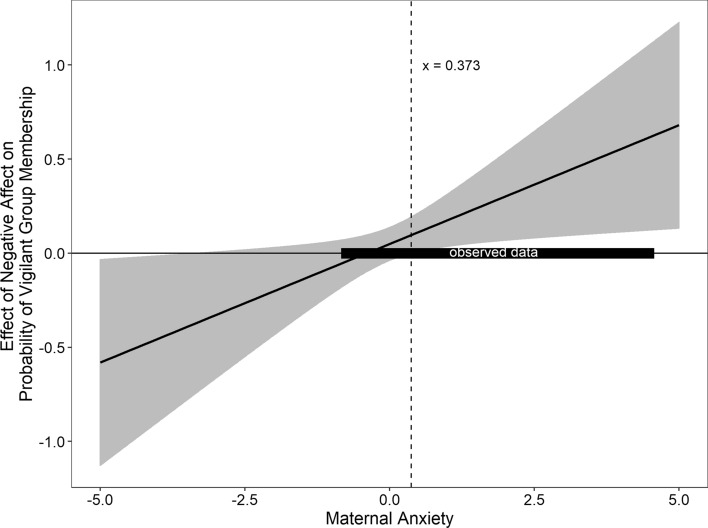
Regions of significance figure probing the moderating role of maternal anxiety on the relation between negative affect and probability of vigilant group membership. The dashed vertical line indicates the point at which the relation becomes significant. The dark horizontal bar represents our observed range of maternal anxiety scores. Results suggest that at higher levels of maternal anxiety and negative affect infants have a higher probability of being in the vigilant group.

To assess how age, negative affect, and maternal anxiety relate to patterns of affect-biased we regressed the three variables and all potential interactions on the probability of *Vigilant* group membership (Table 6). We found a two-way Negative Affect × Maternal Anxiety interaction (*b* = 0.13, *p* = 0.033). Figure 2 displays a Johnsen–Neyman plot created using Preacher Interaction Utilities^41^. The Johnsen–Neyman analysis indicated that at Maternal Anxiety scores ≥ 0.373 (centered; raw score = 5.04) higher levels of Negative Affect were associated with greater probability of being in the *Vigilant* group. We compared this model (BIC = 3230.7) to a model controlling for infant sex and prematurity (BIC = 5030.4), which indicated that the model without covariates was a better fit.

**Table 6:** Regression assessing contributions of age, negative affect and maternal anxiety to probability of vigilant group membership.

	*b*	*β*	*SE*	*z*	*p*
Age	0.01	0.02	0.03	0.24	0.809
Negative affect	0.05	0.09	0.04	1.17	0.243
Maternal anxiety	− 0.03	− 0.07	0.03	− 0.87	0.383
Age × negative affect	− 0.04	− 0.08	0.04	− 1.00	0.318
Age × maternal anxiety	− 0.02	− 0.04	0.04	− 0.46	0.648
Negative affect × maternal anxiety	0.13	0.20	0.06	2.14	0.033
Age × negative affect × maternal anxiety	− 0.01	− 0.02	0.05	− 0.18	0.855

Overall, the relation between age (as a continuous variable) and probability of being in the *Vigilant* group is not significant. However, as noted in the introduction, the five-to-seventh month shift may be a unique developmental window for attention bias. To examine this relation, we split the sample into infants younger (N = 41) and older than 7 months of age. The comparison of probability scores suggests that younger infants may be less likely to be in the *Vigilant* group (0.60 vs. 0.78), *t*(191) = − 2.68, *p* = 0.01.

## Discussion

The overarching goal of the current study was to assess affect-biased attention in infants between 4- and 24-months across three affect-biased attention tasks. In support of this goal, we had two aims (1) to assess affect-biased attention from a variable-centered (factor analysis; FA) approach and a person-centered (latent profile analysis; LPA) approach and (2) to examine how age, negative affect and maternal anxiety related to components of affect-biased attention (variable-centered approach) and groups of infants with a particular type of affect-biased attention (person-centered approach). We found that both a variable-centered approach and a person-centered approach noted specific and distinct relations between affect-biased attention and age, negative affect, and maternal anxiety. To summarize, the exploratory FA found an *Engagement* factor comprising increased dwell to angry and happy faces across the dot-probe and overlap tasks and decreased latency to angry and happy faces in the vigilance task. Additionally, we found a *Disengagement* factor consisting of increased latency to probe in the dot-probe task and increased dwell to the probe in the overlap task.

Thus, across our three tasks, we successfully captured both the engagement and disengagement aspects of orienting that theoretically undergird affect-biased attention^5^. Importantly, our findings highlight the fact that affect biased attention is a multidimensional construct. Often, the literature conflates affect-biased attention with attention bias *towards* threat, although this is simply one form of an attention bias. However, the broader construct encompasses stable patterns of attention both towards and away from salient stimuli, both positive and negative. As such, here we note that maternal anxiety, a risk factor for child anxiety, is associated with less time spent engaging with faces. This finding is consistent with several lines of prior work^8,35^. For example, prior research has reported that a bias away from threat is related to anxiety and fearful temperament in children^17–20^. Recent work also suggests individuals high in social anxiety may have trouble orienting to happy faces^42^.

We also found a main effect of age on our *Disengagement* factor, such that older infants exhibited more difficulty disengaging from stimuli. Dwell to faces and latency to probes within the dot-probe were positively related (Fig. 1), but loaded onto different factors in our FA. Although age did not significantly relate to the *Engagement* factor, the relation between age and *Disengagement* was positive. Typically, we associate age with improving performance, such that infants are expected to be faster and more efficient and more accurate as they complete experimental tasks. Building on this characterization, our a priori expectation was that with greater age infants would exhibit greater attention control, including greater ease disengaging from the presented stimuli. However, chronological age also acts as a proxy for the underlying development of cognitive and perceptual processes that influence how infants engage with their environment. That is, as infants process the faces presented in the task (dwell), they may extract more complex and nuanced information that, in turn, impacts their ability to disengage from the face and orient to the relatively less visually salient probes presented in the tasks.

Our LPA indicated that distinct groups of infants could be identified based on specific patterns of affect-biased attention marked by the clustering of both engagement and disengagement components of orienting. Specifically, we found a *Vigilant* affect-biased attention group that exhibited greater engagement with faces (via longer dwell times to faces across the dot-probe and overlap tasks and shorter latencies to faces in the vigilance tasks) and better disengagement capacities (via shorter latencies to probes in the dot-probe task and shorter dwell times to probes in the overlap task). Our *Avoidant* affect-biased attention group exhibited the reverse (less engagement with faces and more difficulty disengaging). Furthermore, the pattern of greater attention to faces coupled with better disengagement from stimuli in our *Vigilant* group differentiated the LPA from the FA, which found no relation between our *Engagement* and *Disengagement* factors.

Maternal anxiety presented a different relation with attention than seen in the FA. For the LPA, we found that maternal anxiety moderated the relation between negative affect and probability of membership in the *Vigilant* group, such that infants at higher levels of negative affect who also had mothers with greater levels of anxiety exhibited a greater probability of being in the *Vigilant* group. These results are in keeping with theories and data linking maternal anxiety and temperamental negative affect to attention patterns. Again, as noted above, we cannot fully disentangle the genetic and environmental contributors to individual phenotypic profiles^34,36^. However, it appears clear that children who have shared exposure to maternal anxiety and temperamental negative affect are likely to show distinct profiles of affect-biased attention. Across the sample, age was not related to group membership, although there was an indication that the youngest infants were less likely to be in the *Vigilant* group. With the current data we cannot speak to patterns of within individual trajectories of vigilance and avoidance in infancy. Longitudinal work will also allow researchers to see if these profiles act as moderators or mediators of emerging patterns of social withdrawal, social reticence, and social anxiety.

If these patterns hold, it does raise the suggestion that subgroups of children would have markedly different interactions with the environment. In particular, children in the *Vigilant* group may receive more social input, if their behavior with faces in the presented tasks carries over into their daily attention patterns. Within the context of social information processing models^43^, these children may have more data to process, interpret and react to faces. This information, in the context of either maternal anxiety, negative affect, or both may generate profiles of social avoidance. Mobile eye-tracking technology^44,45^, may help determine if visual attention patterns evident in computer-based tasks also carry over to dynamic social interactions.

These arguments regarding the potential down-stream impact of attention echoes prior discussion in the anxiety risk literature incorporating measures at multiple levels of analysis. For example, the frontal EEG alpha asymmetry literature suggests that patterns of approach, linked to left frontal asymmetry, and avoidance, linked to right frontal asymmetry, motivation are reflected in anxiety risk^46,47^. The attachment literature, in turn, suggests that profiles marked by secure relationships are associated with greater engagement with the environment, building on the availability of a safe base as needed^48^. Thus, there is a conceptual thread in both the developmental and clinical literature suggesting that broad patterns of exploration (versus exploitation) of the environment are associated with socioemotional functioning^49^. However, few studies have captured the presumed temporal and contextual ripple effects from basic attentional processes to socioemotional profiles. One such study^50^ found that increased attentional bias to fearful faces at 7 months of age predicted secure infant–mother attachment in the strange situation at 14 months. In contrast, a smaller bias was associated with insecure attachment and attachment disorganization was linked to the absence of an attentional bias to fear.

The attention bias literature, as a whole, has been hampered by the over-reliance on a single task—the dot-probe—particularly with respect to reaction-time based difference scores. Prior work has shown poor reliability^51^ and two general recommendations have emerged from critiques of the literature. First, multiple tasks should be used to calculate bias, and, ideally the tasks should be repeated within the same individual in order to generate more stable and robust patterns of attention^52^. Second, the literature should move away from reaction time-based difference scores, as they can exacerbate measurement error^53^. On this point, recent reviews focused on adults^54^ and children^10^ suggest that measures more proximal to the processing of salient stimuli, captured via functional magnetic resonance imaging (fMRI), event-related potentials (ERPs), or eye-tracking have more robust psychometric properties. In the current study, the use of eye-tracking across multiple measures incorporates both recommendations, while having the added bonus of being developmentally appropriate for use starting in the first months of life.

The current study is limited by the cross-sectional nature of the data and our linear methods for analyzing relations between affect-biased attention and age. Previous work indicates there might be a normative increase in threat detection during infancy between 5- and 7-months^24^ that then tapers off^25^. Our cross-sectional data limit our ability to examine potential curvilinear relations between age and affect-biased attention. Longitudinal samples might enable the detection of distinct trajectories of affect-biased attention. For example, infants with anxious mothers and high levels of negative affect may exhibit stable or increasing affect-biased attention with age, whereas infants lower in negative affect or who do not have anxious mothers may be more likely to show transient or moderate patterns of attention bias over time. Thus, our current work should not be taken as evidence that developmental change is not an aspect of affect-biased attention but rather be considered as a first step to testing questions regarding the emergence of affect-biased attention across the first two years of life.

We focused on maternal traits of self-reported anxiety, which limits insights into the multiple mechanisms that can shape developmental profiles. As noted above, without a genetically-informed design, we cannot parse the contribution of shared genetic risk for anxiety or affect bias, from the impact of daily interactions and socialization. In addition, we did not incorporate information from the other genetically-related parent, nor other caregivers in the daily life of the children. Again, this limits the extent to which we can capture potentially causal mechanisms that shape patterns of negative affect and attention over the course of infancy.

In addition, we highlight that both the person- and variable-centered approaches illustrated here are dependent on the specific data used in our models. We implemented a data-driven approach as we wished to illustrate in this initial study that variations in our understanding of how individual differences and environmental factors relate to affect-biased attention might emerge based on how affect-biased attention is modeled. This is in contrast to first proposing specific profiles and factors decided a priori that are then tested with the data. As such, variation in study population, with respect to age, anxiety risk, or other characteristics, coupled with variation in the specific tasks used, could result in a different constellation of outcomes. A larger sample of older children, for example, may present with greater variability in task performance leading to more than two factors or profiles. Older children are also likely to show more complex and heterogeneous socioemotional profiles. Analytic techniques designed to assess change over time, such as Latent Transition Analysis (LTA)^55^ may capture how children transition between patterns of affect biased attention with development and as a function of individual differences and contextual factors.

The current study examined both variable-centered and person-centered approaches for understanding profiles and patterns of affect-biased attention. We found that both approaches were informative, revealing distinct relations between metrics of affect-biased attention in early life. In our variable-centered approach, we found that higher levels of maternal anxiety were related to less engagement with emotional faces. Additionally, we found that greater age was related to more difficulty with disengagement from emotional faces. In contrast, our person-centered approach found a group of infants that exhibited both more attention to faces and more ease with disengagement. Infants were more likely to be members of this group when they exhibited higher levels of negative affect and had more anxious mothers. The current analyses provide insight into (1) ways of measuring affect-biased attention across multiple tasks and (2) how individual difference factors relate to affect-biased attention when modeled in variable-centered versus person-centered approaches. Furthermore, the current results set the stage for future longitudinal work examining how affect-biased attention develops over time in the context of both infant negative affect and maternal anxiety.

## Method

### Study overview

We recruited families with infants between the ages of 4- and 24-months to participate in a larger study examining the relations between affect-biased attention and temperament. Prior to the laboratory visit, mothers rated their levels of anxiety and their infant’s temperament. At the laboratory visit, infants completed three infant appropriate stationary eye-tracking tasks as well as a behavioral battery to assess temperament. Approval for this study, titled Visual Attention and Behavior in Infants, was granted by The Pennsylvania State University Institutional Review Board (IRB) with study number PRAMS0004009. All methods were carried out in accordance with the relevant guidelines and regulations of the IRB. Parents provided informed consent for both their own and their infant’s participation. Families were compensated for their participation. Data are accessible through Databrary^56^ for those participants who consented to data sharing.

### Participants

The final sample for the current analyses consisted of 193 infants (*M*_age_mo_ = 12.34, *SD*_age_mo_ = 5.67, *Range*_age_mo_ = 4.00 to 24.30) drawn from the 261 infants who participated in the larger study. We selected these 193 infants for inclusion as each provided some usable eye-tracking data across one of the three eye-tracking tasks (see Affect Biased Attention Measures and S1). A-priori power analyses and previous literature indicated our 193 infants were sufficient for our planned analyses (S2). Previous publications presented analyses of the individual tasks^7–9,57^. However, the current analyses are unique in that they bring all three tasks together.

Participants were recruited via mailings sent to parents identified using a university-based database of families interested in research, as well as community advertisements. The initial sample was predominantly White (92.7%), reflecting the surrounding semi-rural community. The remaining 7.3% of families self-identified as Asian-American, African-American, Native-American or Hispanic. All families reported that English was spoken at home, while 23 infants were also exposed to a second language. All children, except two, were living with a biological parent. Infants had adequate birth weight (*M*_weight_lbs_ = 7.64, *SD*_weight_lbs_ = 1.13). Eleven infants (5 male) were born more than three weeks prior to their due date. We calculated the difference between due date and actual birth date for the sample (*M*_days_ = -3.04, *SD*_days_ = 9.76) and found no relations with our variables of interest, *p*’s > 0.10. Families reported that infants were meeting motor milestones (rolling over, crawling, and walking) within normal developmental windows. Age of milestones was not associated with task variables, *p*’s > 0.24.

### Affect-biased attention measures

The general protocol for eye-tracking data collection has been published in our previous work^7–9^. Infants in the study were presented with three eye-tracking tasks (dot-probe, overlap, vigilance) designed to assess complementary, but not identical, components of affect-biased attention. All three tasks used faces (Angry, Happy and Neutral) taken from the NimStim face stimulus set^58^. Eye-tracking data were obtained using a RED-m Eye Tracking System (SensoMotoric Instruments) and an integrated 22-inch presentation monitor (8.5 cm by 6.3 cm screen). Infants were seated 60 cm from the monitor on either an adjustable highchair, or their parent’s lap, such that their eye gaze was centered on the screen. Seating position did not differ across tasks (27.5% in the dot-probe task, 27.9% in the overlap task, and 23.7% in the vigilance task). We found no relation between seating arrangement and core study measures (*p*’s > 0.11), except that for the dot-probe task, infants on the lap were younger than infants in the high chair (*p* = 0.02).

The eye-tracker monitor has cameras embedded that record the reflection of an infrared light source on the cornea relative to the pupil from both eyes, which enables tracking of eye-movements. The average accuracy of this eye-tracking system is in the range of 0.5°–1°, which approximates to a 0.5–1 cm area on the screen with a viewing distance of 60 cm. The testing procedure began with a 5-point calibration and four-point validation procedure using an animated multicolored circle. Testing continued until all trials had been presented, or the infant’s attention could no longer be maintained. Gaze information was sampled at 60 Hz and collected by Experiment Center (SensoMotoric Instruments, Teltow, Germany).

As recommended^59^, trials in each task were triggered by infant fixation rather than predetermined presentation timings. Each trial began with a central fixation (a clip from a children’s movie), which was presented until the infant fixated for at least 100 ms. Task-specific areas of interest (AOIs) were created using BeGaze (SensoMotoric Instruments). Fixation locations and durations within the AOIs were calculated for each trial with in-house Python (Python Software Foundation, http://www.python.org/) and MATLAB (The MathWorks, Inc., Natick, Massachusetts, USA) scripts.

When an infant was unable to complete the full eye-tracking and temperament protocol in a single day, they would return for a second visit (N = 80; 30.5%, *M*_*visit_gap*_ = 5.60 days, *SD*_*visit_gap*_ = 5.86). In these cases, we attempted to complete any tasks not completed in the first visit. Neither the need for a second visit, nor the length of the gap, was associated with study variables, *p*’s > 0.09.

#### Infant dot-probe task

The general protocol for the dot-probe task has been published in our previous work^9^. The dot-probe task consisted of 30 experimental trials. Three types of face pairs were included: angry-neutral (6 congruent trials, 6 incongruent trials), happy-neutral (6 congruent trials, 6 incongruent trials), and neutral–neutral (6 trials). There were 6 faces used (3 male), all presented once in each face-pair type. The face pictures were each 14.0 cm × 19.0 cm and were presented side-by-side, with a distance of 26.5 cm between their centers.

Given the infant sample in the current study, faces were presented for 1000 ms, providing sufficient time to capture eye-gaze patterns for even the youngest participants. Faces were then removed and immediately replaced by a probe (a black asterisk centered on a white screen), which remained on screen for 500 ms. The inter-trial interval was 1000 ms. Shorter presentation times used in other versions of the task^22^ capture more automatic processing. The extended presentation time in our task captures engagement with (dwell to faces) and disengagement from (latency to probes) faces^9^.

AOIs encircled and included the entire face and probe display areas and fixations were defined as gaze maintained for at least 80 ms within a 100-pixel maximum dispersion. Dwell time for both angry and happy faces as well as latency to orient to the probe during angry-neutral and happy-neutral trials were extracted for analyses.

#### Overlap task

The general protocol for the overlap task has been published in our previous work^8^. The overlap task consisted of 12 experimental trials. A face appeared on the screen for 1000 ms followed by the distractor, which appeared together with the face for 3000 ms. The distractor consisted of a static black-and-white checkerboard patterned rectangle that appeared vertically oriented on the edge of either the left or right side of the screen (counterbalanced). Twelve faces were used (6 male). The face pictures were each 11.8 cm × 8.5 cm, the distractor was 12.0 cm × 2.0 cm with a distance of 22.5 cm between their centers. AOIs delineated the top, bottom, and contour of the face and probe locations. Fixations were defined as gaze maintained for at least 80 ms within a 100 pixel maximum dispersion, were extracted with BeGaze. Dwell time to the angry and happy faces during overlap trials (probe present; capturing engagement with faces) as well as dwell time to the probe (capturing disengagement from faces) were extracted for analyses.

#### Vigilance task

The general protocol for the vigilance task has been published in our previous work^7^. The task consisted of 45 trials. A face appeared in one of the four corners of the computer screen. Ten actors (5 male) provided neutral, angry and happy facial expressions. Each category of facial expression was presented for 15 trials. No individual face appeared in the same location more than once. The face pictures were each 5.08 cm × 3.68 cm. Each trial advanced after 100 ms fixation on the target face or after 4000 ms if no fixation was detected. Every 7 trials, a blank white screen was presented for 4000 ms. The order of face stimulus was randomized across participants. Latencies to orient to the angry and happy faces (capturing initial engagement with faces) were extracted for analyses.

### Individual differences measures

#### Infant negative affect

We assessed infant negative affect were assessed via both maternal report (IBQ-R^60^, TBAQ^61^) and direct observation of behavior (Reactivity^62^, Lab-TAB^63^; see S3 for full protocol). Neither laboratory procedures nor parental observations independently capture the full range of a child’s behavior^64–66^. This is seen in our own data where maternal report of negative affect and observed negative affect were related at *r* = 0.19, *p* = 0.24. This relation reveals an overlap between measurements while highlighting the unique information to be gleaned from both reported and observed data^67^. Thus, we created a “risk score” of negative affect by averaging standardized maternal report and laboratory observation measures for the full sample (procedure below). Infants with only one negative affect score (maternal report or laboratory observation; *N* = 21) were retained in the analyses, creating a final sample of 260 infants (*M*_*NA*_ = -0.001, *SD*_*NA*_ = 0.77).

#### Standardized maternal report of infant negative affect

Infants characterized by the IBQ-R and TBAQ did not differ in sex, birth-weight, or other demographics (*p*’s > 0.29), except for the presence or absence of age-linked motor milestones. Individual scores from each questionnaire were standardized (*Range*_*IBQ-R_NA*_ = − 2.20 to 3.25; *Range*_*TBAQ_NA*_ = − 2.03 to 3.10) and merged into a single Negative Affect measure (*N* = 252, *M*_*NA*_ = 0.00, *SD*_*NA*_ = 1.00).

#### Standardized observed infant negative affect score

Infants assessed by the two laboratory batteries did not differ in sex, birth weight, the difference between birth date and due date, or other demographic measures (*p*’s > 0.31), except for the presence or absence of age-related motor milestones. We created an observed negative affect score by standardizing the negative affect scores for 4- to 8-month and 8- to 24-month infants (*Range*_*NA_4-8mo*_ = − 0.75 to 3.61; *Range*_*NA_8-24mo*_ = − 1.85 to 4.27), respectively, and then merging the standardized scores into a single observed NA measure (overall sample: *N* = 250, *M*_*NA*_ = 0.00, *SD*_*NA*_ = 1.00). Outlier negative affect composite scores (> M + 3SD) were excluded from data analyses (final sample: *N* = 247, *M*_*NA*_ = -0.03, *SD*_*NA*_ = 0.94).

#### Maternal anxiety

Mothers (*N* = 223) completed the Beck Anxiety Inventory (BAI)^68^, a 21-item self-report scale that measures anxiety symptoms during the past month. Each item is rated on a 4-point scale from 0 (“Not at all”) to 3 (“Severely”). In the current sample, the measure had adequate reliability (Cronbach’s α = 0.88). Mothers presented with a wide range of scores (*Mean*_BAI_ = 4.96, *SD*_BAI_ = 5.89, *Range*_BAI_ = 0–38) with 171 (76.7%) in the healthy range, 38 (17.0%) in the elevated range, and 14 (6.3%) in the clinical range.

### Data analysis

Our first aim was to examine affect-biased attention across three tasks using both a variable-centered (FA) and a person-centered (LPA) approach. The FA was conducted in the R package lavaan^69^. Missing data were handled within lavaan using FIML. The LPA was conducted in the R package mclust^39^ which fits Gaussian finite mixture models using an expectation–maximization (EM) algorithm^70^. Missing data were handled within the mclust package using the mix package, which computes maximum-likelihood estimates for the parameters of the unrestricted general location model^71^. We included the same eye-tracking metrics in both models. Prior to inclusion in the models, all variables were scaled from milliseconds to seconds by dividing by 1000.

Our second aim was to examine if individual difference factors known to relate to affect-biased attention (age, negative affect, and maternal anxiety) related to the factors (FA) and profiles (LPA). To do so, we examined the FA in a Structural Equation Model (SEM) framework and the LPA in a multiple regression framework, both in lavaan using FIML to address missing data. In both cases, we included age, negative affect, and maternal anxiety, as well as all potential interactions as predictors. We also compared these models to separate models that included sex and prematurity as covariates.

## Supplementary Information

Supplementary Information.

## Data Availability

The data that support the findings of this study are openly available in Databrary at https://nyu.databrary.org/volume/119^56^.
