# Supplementary material for: Variable- and person-centered approaches to affect-biased attention in infancy reveal unique relations with infant negative affect and maternal anxiety
Source: Sci Rep. 2021 Jan 18;11:1719. doi: 10.1038/s41598-021-81119-5 (PMC7814017; doi:10.1038/s41598-021-81119-5)
Supplement: Supplementary file 1 — Supplementary Information. [file 41598_2021_81119_MOESM1_ESM.docx]

Variable- and Person-Centered Approaches to Affect-Biased Attention in Infancy Reveal Unique Relations with Infant Negative Affect and Maternal Anxiety

Alicia Vallorani^1*^, Xiaoxue Fu^2^, Santiago Morales^3^,

Vanessa LoBue^4^, Kristin A. Buss^1^ & Koraly Pérez-Edgar^1^

^1^The Pennsylvania State University, University Park, PA

^2^University of South Carolina, Columbia, SC

^3^University of Maryland, College Park, MD

^4^Rutgers University, Newark, NJ

^*^Corresponding author

The study was supported by grants from the National Institute of Mental Health to Dr. Koraly Pérez-Edgar (R21MH103627), to Drs. Koraly Pérez-Edgar, Kristin Buss, and Vanessa LoBue (R01MH109692) and to Alicia Vallorani (F31 MH121035).

Correspondence concerning this article should be addressed to Alicia Vallorani, Department of Psychology, The Pennsylvania State University, University Park, PA 16802. Contact: auv27@psu.edu

The data that support the findings of this study are openly available in Databrary at https://nyu.databrary.org/volume/119 ^1^.

**Supplement**

**S1. Selecting Useable Eye-Tracking Data**

Trial data were extracted and processed using in-house Python (Python Software Foundation, https://www.python.org/) and MATLAB (The MathWorks, Inc., Natick, Massachusetts, USA) scripts. Data processing procedures were set to maximize the quantity and quality of the data available for subsequent analyses. This is in line with the infant literature’s ^2–4^ concern for designing tasks that are amenable for infant use, provide a rich yet reliable data set, and balance concerns regarding Type I and Type II error. As such, we had a multi-step process that spanned from data collection to data processing, excluding infants or data points that were likely to introduce excessive noise.

If infants were overly distressed or deemed unable to attend to the procedures or were unable to calibrate at the start of the task, we did not attempt the specific task. Of the 261 infants enrolled in the larger study, 240 attempted the dot-probe task, 163 attempted the overlap task, and 230 attempted the vigilance task.

Second, if the infant stopped attending during the first-half of the task, he/she was designated as not having completed the task (*N* = 65 for dot-probe, *N* = 28 for overlap, *N* = 32 for vigilance). If an infant made it past the halfway point of the task, infants were designated as having completed the task.

Finally, we assessed the quality of the collected data post-visit by examining the calibration measures. We specifically focused on the average deviation of the infant’s eye gaze relative to the location of the 5 calibration points, in line with reviews suggesting that initial calibration is crucial to providing robust and reliable data ^5^. If the deviation of the coordinates in the X or Y direction was greater than four degrees, the child was excluded from further processing (*N* = 24 for dot-probe, *N* = 7 for overlap, *N* = 17 for vigilance).

Once an infant met each criterion, all data provided by the infant were deemed available for analyses (*N* = 148 for dot-probe, *N* = 128 for overlap, *N* = 161 for vigilance). Across the sample, 112 infants provided data for all three tasks, 38 for two tasks, and 43 for one task. In aggregate, 193 infants provided data for at least one task. Although published studies vary widely on inclusion/exclusion criteria, the final yield is normative for laboratory studies in this age range ^6^.

For our analyses, infants provided on average 23.7 trials (*SD*_trials_ = 7.25, Range = 1 to 30) in the dot-probe task, 11.3 trials (*SD*_trials_ = 1.53, Range = 4 to 12) in the overlap task, and 28.3 trials (*SD*_trials_ = 12.38, Range = 1 to 45) in the vigilance task.

**S2. A Priori Power Analyses**

*Factor Analysis and Structural Equation Model*

Using Computing Power and Minimum Sample Size for RMSEA ^7^, we determined a sample size of at least 189 infants was needed to achieve an RMSEA of < .08 (good fit) with 16 predictors. Thus, we are underpowered when examining the factor analysis independently. However, we are adequately powered at 193 infants to assess the SEM model as a whole.

*Latent Profile Analysis*

Little work has investigated the power necessary to conduct latent profile analysis (LPA), likely because the null model is not clearly defined in this particular method ^8^. Work by Wurtps and Geiser ^9^ examining latent class analysis, the dichotomous variable companion to LPA, suggests there is an important relation between sample size, number of indicators in the model, and quality of indicators in the model, that supports power to detect latent classes. Specifically, a larger sample (≥ 300) is better generally speaking. However, with multiple, strong indicators (high quality, 5+ indicators), smaller Ns (≥ 100) are tenable. Our LPA includes 193 infants and 10 high quality indicators, suggesting we have the power necessary to conduct our analysis.

*Regression Model*

Using G*Power 3.1.9.4 ^10^, we determined that assessing a medium effect (0.15) using a multiple regression with 7 predictors at a power of .8 and an alpha level of .05 requires a sample size of at least 103 infants. Thus, our sample size of 193 infants was sufficient to detect moderate effect sizes.

**S3. Protocols for Assessing Infant Temperamental Negative Affect**

*Maternal Report of Infant Temperamental Negative Affect*

Infant Behavior Questionnaire-Revised (IBQ-R). Parents of 4- to 12-month-old infants (*N* = 147, 83 boys, *M_age_mo_* = 7.22, *SD_age_mo_* = 2.44) completed the IBQ-R ^11^, a 191-item questionnaire that assesses the frequency of specific infant behaviors during the previous week. Parents rated the frequency of behaviors using a seven-point scale with an eighth option for “Does not apply.” Subscales were derived by taking the mean ratings of all items for each subscale, omitting the items marked as “Does not apply.” Of particular interest in this study was the negative affect subscale, which assesses sadness, distress to limitations, fear, and reactivity/recovery (*M_IBQ-R_NA_* = 3.61, *SD_IBQ-R_NA_* = 0.48, Cronbach’s α = .81).

Toddler Behavior Assessment Questionnaire (TBAQ). Parents of infants between the ages of 12- and 24-months (*N* = 105, 57 boys, *M_age_mo_* = 16.96, *SD_age_mo_* = 4.06), completed the TBAQ ^12^, a 120-item rating form that assesses frequency of specific infant behaviors during the previous month. The TBAQ is modeled after the IBQ and uses a similar response format—a seven-point Likert scale with an eighth option for “Does not apply.” Subscales were derived by taking the mean ratings of all items for each subscale, omitting the items marked as “Does not apply.” Of particular interest in this study was the NA subscale, made up of items assessing sadness, distress to limitations, fear, and reactivity/recovery (*M_TBAQ_NA_* = 3.23, *SD_TBAQ_NA_* = 0.58, Cronbach’s α = .82).

*Observed Infant Temperamental Negative Affect*

Infants 4- to 8-months of age (*N* = 97, 53 boys, *M_age_mo_* = 5.89, *SD_age_mo_* = 1.10) completed a standard battery of novel auditory and visual stimuli to assess temperamental reactivity ^13–15^. Two blocks of stimulus presentation began with infants seated in a car seat in a quiet and alert state. Each block of stimuli consisted of a set of alternating auditory and visual presentations. The order of the two auditory and visual presentations was counterbalanced across participants.

One of the auditory presentations contained nonsense syllables (ma, ga, pa). Each syllable was presented in three consecutive 10-second trials, with 5-second inter-trial intervals (ITIs). The other auditory presentation consisted of 8 sentences, each lasted about 6-second in duration with 2-second ITIs. The sentences were presented in 4 pairs, which differed in the number of voices speaking together (one, two, three, and four voices). One of the visual presentations consisted of mobiles differing in the number of hanging stuffed jungle animals (one, three, five). Each mobile was displayed for 20 seconds (ITIs were approximately 10 seconds) above the infant’s face at an unreachable distance. The other visual presentation followed the same procedure, except that the elements on the mobiles were stuffed bears. All sessions were videotaped, allowing for behavioral coding of infant reactivity.

Coding was based on previously described procedures ^13–15^. Prorated duration scores were computed for all behaviors of interest to account for infants who did not complete the full procedure. Infant temperamental NA was computed by taking the sum of prorated duration scores of fussing and crying scores (*M_NA_4-8mo_* = 15.26, *SD_NA_4-8mo_* = 20.41). Interrater reliability was calculated on ~20% of the data with 91.8% agreement and κ = 0.57.

Infants between 8- and 24-month of age (*N* = 151, 84 boys, *M_age_mo_* = 14.59, *SD_age_mo_* = 4.67) completed six episodes adapted from the Laboratory Temperament Assessment Battery ^16^ to assess temperamental fear, joy, and anger/frustration. The episode order was constant across participants and was as follows: *Stranger Approach*, *Puppet Show*, *Container*, *Peek-a-boo*, *Unpredictable Mechanical Toy* and *Gentle Arm Restraint*. The procedure began with infants seated in a high chair in a neutral and alert state. Episodes were terminated if the infant became overly distressed.

Two episodes, *Unpredictable Mechanical Toy* and *Stranger Approach*, were designed to elicit fear. In *Unpredictable Mechanical Toy*, 8- to 15-month-old infants were presented with a mechanical dog that moved towards the infant 3 times with 10-second pauses in between movements. The dog barked after the final approach and then the experimenter invited the child to play with the object. For 15- to 24-month-old infants, a large plush spider approached the infant and then retreated twice with 10-second pauses in between movements. Both paradigms ended with the experimenter inviting the child to play with the object. For the *Stranger Approach* episode, a male research assistant entered the room, walked towards the infant slowly, pausing for 15 seconds at two predesignated stopping points.

*Puppet Show* and *Peek-a-boo* were designed to elicit joy. During the *Puppet Show* episode, the experimenter presented a puppet show. After the show ended, the researcher invited the infant to interact with the puppets. For *Peek-a-boo*, the experimenter sat behind a curtain screen, and intermittently revealed his or her face for 2 seconds and said “peek-a-boo”. The game continued for 6 trials.

*Container* and *Gentle Arm Restraint* assessed anger/frustration. In *Container*, infants played with an attractive toy. The experimenter then took the toy away and placed it in a closed, transparent container for 30 seconds. The trial was repeated two more times. The infant played with the toy between trials. In *Gentle Arm Restraint*, the infant played with an attractive toy. The parent then held down the infant’s forearms so the infant could not reach the toy for 30 seconds. The procedure was repeated a second time. The infant played with the toy between trials.

Coding was based on previously described procedures ^16^. Infant negative affect was computed by summing the total intensity of facial anger, facial sadness, bodily sadness, distress vocalization, and struggle across all six episodes (*M_NA_8-24mo_* = 59.88, *SD_NA_8-24mo_* = 30.31). Interrater reliability was calculated on ~20% of the data with 90.3% agreement and κ = 0.92.

**S4. Task performance as a function of age**

Overall, we noted a relation between number of trials per experiment and age for the dot-probe, *r* = .17, *p* = .04, and vigilance, *r* = .18, *p* = .02, tasks, but not the overlap task, *r* = .13, *p* = .16. A systematic set of studies ^17–19^ have noted that infants show distinct shifts in attention bias at or around 7 months of age. As noted in the main text, younger infants were less likely to be in the *Vigilant* group. Here we provide additional information regarding the sample split based on age.

With respect to their overall contribution to the sample, 41 of the 193 infants (21%) were less than 7 months of age. In comparison, 82 of the 261 infants enrolled (31%) were less than 7 months of age. Across tasks, the younger infants provided fewer trials in the vigilance task (24.0 vs. 29.4, *t* = -2.26, *p* = .02), but did not differ on the dot-probe (20.7 vs. 24.3, *t* = -1.85, *p* = .07) and overlap (10.5 vs. 11.4, *t* = -1.64, *p* = .11) tasks.

*Table S1: Descriptive Statistics for Eye-Tracking Metrics of Interest*

|  | *M* | *SD* | N |  |
| --- | --- | --- | --- | --- |
| DP: Dwell Time to Angry Faces | 0.42 | 0.14 | 139 |  |
| DP: Dwell Time to Happy Faces | 0.41 | 0.15 | 143 |  |
| DP: Latency to Probe in Angry Trials | 0.30 | 0.08 | 114 |  |
| DP: Latency to Probe in Happy Trials | 0.29 | 0.09 | 114 |  |
| OV: Dwell Time to Angry Faces | 1.32 | 0.67 | 126 |  |
| OV: Dwell Time to Happy Faces | 1.34 | 0.68 | 125 |  |
| OV: Dwell Time to Probe in Angry Trials | 0.48 | 0.37 | 103 |  |
| OV: Dwell Time to Probe in Happy Trials | 0.42 | 0.27 | 103 |  |
| VI: Latency to Angry Faces | 0.56 | 0.29 | 155 |  |
| VI: Latency to Happy Faces | 0.54 | 0.24 | 157 |  |

*Note*: DP = dot-probe task, OV = overlap task, VI = vigilance task;

*M* = mean, *SD* = standard deviation

*Table S2.* *Fit Statistics for 2- and 3-Factor Models*

*Assessing Affect-Biased Attention Across Tasks*

|  | 2-Factor | 3-Factor |  |
| --- | --- | --- | --- |
| Chi-Square | 44.50 | 40.68 |  |
| CFI | 0. 95 | 0. 96 |  |
| BIC | -190.83 | -184.12 |  |
| RMSEA | 0.042 | 0.040 |  |
| SRMR | 0.077 | 0.068 |  |

*Table S3.* *Fit Statistics for 1-, 2- 3- and 4-Profile Models*

*Assessing Affect-Biased Attention Across Tasks*

|  | 1-Profile | 2-Profile | 3-Profile | 4-Profile |  |
| --- | --- | --- | --- | --- | --- |
| Loglikelihood | 352.08 | 459.26 | 492.81 | 512.74 |  |
| BIC | 362.09 | 465.93 | 422.50 | 351.86 |  |
| AIC | -574.17 | -746.52 | -771.61 | -769.48 |  |
| BLRT |  | 214.36* | 67.09* | 39.87 |  |

**p* < .05

References

1. Pérez-Edgar, K. A-n-T: Attention and Temperament in Infancy. *Databrary* https://nyu.databrary.org/volume/119 (2015).

2. Leppänen, J. M. Using eye tracking to understand infants’ attentional bias for faces. *Child Dev. Perspect.* **10**, 161–165 (2016).

3. Oakes, L. M. Advances in eye tracking in infancy. *Infancy* **17**, 1–8 (2012).

4. Oakes, L. M. Sample Size, Statistical Power, and False Conclusions in Infant Looking-Time Research. *Infancy* **22**, 436–469 (2017).

5. Morgante, J. D., Zolfaghari, R. & Johnson, S. P. A critical test of temporal and spatial accuracy of the Tobii T60XL eye tracker. *Infancy* **17**, 9–32 (2012).

6. Stets, M., Stahl, D. & Reid, V. M. A meta-analysis investigating factors underlying attrition rates in infant ERP studies. *Dev. Neuropsychol.* **37**, 226–252 (2012).

7. Preacher, K. J. & Coffman, D. L. *Computing power and minimum sample size for RMSEA*. (2006).

8. Tein, J.-Y., Coxe, S. & Cham, H. Statistical power to detect the correct number of classes in latent profile analysis. *Struct. Equ. Model. Multidiscip. J.* **20**, 640–657 (2013).

9. Wurpts, I. C. & Geiser, C. Is adding more indicators to a latent class analysis beneficial or detrimental? Results of a Monte-Carlo study. *Front. Psychol.* **5**, (2014).

10. Faul, F., Erdfelder, E., Lang, A.-G. & Buchner, A. G*Power 3: A flexible statistical power analysis program for the social, behavioral, and biomedical sciences. *Behav. Res. Methods* **39**, 175–191 (2007).

11. Gartstein, M. A. & Rothbart, M. K. Studying infant temperament via the revised infant behavior questionnaire. *Infant Behav. Dev.* **26**, 64–86 (2003).

12. Goldsmith, H. H. Studying Temperament via Construction of the Toddler Behavior Assessment Questionnaire. *Child Dev.* **67**, 218–235 (1996).

13. Calkins, S. D., Fox, N. A. & Marshall, T. R. Behavioral and physiological antecedents of inhibited and uninhibited behavior. *Child Dev.* **67**, 523–540 (1996).

14. Fox, N. A., Henderson, H. A., Rubin, K. H., Calkins, S. D. & Schmidt, L. A. Continuity and discontinuity of behavioral inhibition and exuberance: Psychophysiological and behavioral influences across the first four years of life. *Child Dev.* **72**, 1–21 (2001).

15. Kagan, J. & Snidman, N. Temperamental factors in human development. *Am. Psychol.* **46**, 856–862 (1991).

16. Buss, K. A. & Goldsmith, H. H. Manual and normative data for the Laboratory Temperament Assessment Battery--Toddler Version. (2000).

17. Peltola, M. J., Leppänen, J. M., Palokangas, T. & Hietanen, J. K. Fearful faces modulate looking duration and attention disengagement in 7-month-old infants. *Dev. Sci.* **11**, 60–68 (2008).

18. Peltola, M. J., Leppänen, J. M., Mäki, S. & Hietanen, J. K. Emergence of enhanced attention to fearful faces between 5 and 7 months of age. *Soc. Cogn. Affect. Neurosci.* **4**, 134–142 (2009).

19. Peltola, M. J., Hietanen, J. K., Forssman, L. & Leppänen, J. M. The emergence and stability of the attentional bias to fearful faces in infancy. *Infancy* **18**, 905–926 (2013).
